# Cannabidiol- and Celecoxib-Loaded Liposomes as a Strategy to Modulate Redox and Inflammatory Signaling in High-Grade Glioma: A Preliminary In Vivo Study

**DOI:** 10.3390/ijms27146220

**Published:** 2026-07-12

**Authors:** Anna Rybarczyk, Aleksandra Majchrzak-Celińska, Ludwika Piwowarczyk, Szymon Tomczak, Dorota Wronka, Anna Karlik, Łukasz Przybył, Violetta Krajka-Kuźniak

**Affiliations:** 1 Poznan University of Medical Sciences, Department of Pharmaceutical Biochemistry, Rokietnicka 3, 60-806 Poznań, Poland; anna.rybarczyk@student.ump.edu.pl (A.R.); majchrzakcelinska@ump.edu.pl (A.M.-C.); 2 Poznan University of Medical Sciences, Doctoral School, Bukowska 70, 60-812 Poznań, Poland; 3 Poznan University of Medical Sciences, Department of Pharmaceutical Chemistry, Rokietnicka 3, 60-806 Poznań, Poland; lpiwowarczyk@ump.edu.pl (L.P.); szymon.tomczak@ump.edu.pl (S.T.); 4 Polish Academy of Sciences, Institute of Bioorganic Chemistry, Laboratory of Mammalian Model Organisms, Noskowskiego 12/14, 61-704 Poznań, Poland; dwronka@ibch.poznan.pl (D.W.); akarlik@ibch.poznan.pl (A.K.); lukasz.przybyl@ibch.poznan.pl (Ł.P.)

**Keywords:** high-grade glioma, cannabidiol (CBD), celecoxib (CELE), nanoformulations, liposomes, combinatorial treatment, NF-κB pathway, Nrf2 pathway, Wnt/β-catenin pathway, mice xenograft model

## Abstract

Inflammation contributes to the rapid progression of high-grade gliomas, indicating that anti-inflammatory strategies targeting NF-κB signaling may offer therapeutic benefit. Cannabidiol (CBD) and celecoxib (CELE) are hydrophobic pharmacological agents whose formulation in lipid carriers may support their combined biological evaluation. In this proof-of-concept study, we investigated liposomal formulations containing CBD, CELE, or both compounds in U-87 MG high-grade glioma cells and in a subcutaneous xenograft model. We assessed cytotoxicity, apoptosis, oxidative stress, Nrf2-dependent responses, NF-κB-centered inflammatory networks, tumor cell invasive properties, and Wnt/β-catenin pathway activity. The nanoformulations induced reactive oxygen species generation by 1.8-fold, which was accompanied by Nrf2 activation. Cationic formulations loaded with the compounds produced more pronounced pro-apoptotic effects (up to 39%) than POPC liposomes, although both types reduced the nuclear translocation of the NF-κB p65 subunit. The CBD + CELE-containing formulation showed a trend toward reduced tumor progression in mice. It is important to note that the in vitro and in vivo nanoformulations were physicochemically related, but not identical, and the in vivo experiment should be interpreted as a preliminary assessment after intratumoral administration. Overall, cationic liposomes co-loaded with CBD + CELE represent a promising platform for further optimization aimed at coordinated modulation of inflammatory, oxidative, and proliferative pathways in glioma. However, additional studies, including tissue distribution, release kinetics, and efficacy in orthotopic glioma models, are needed to fully verify their translational potential.

## 1. Introduction

As the predominant primary tumors of the brain, gliomas account for the highest rates of mortality among central nervous system (CNS) cancers [1,2]. Despite highly invasive treatments, including surgical resection, adjuvant chemoradiotherapy, and advanced technologies such as tumor-treating fields (TTFs), high-grade glioma outcomes remain limited, with recurrence reported in 75–90% of cases [3,4,5]. The primary challenge in treating gliomas is the blood–brain barrier (BBB)-mediated limitation of drug delivery together with intratumoral heterogeneity that promotes tumor cell survival and therapeutic resistance [6,7].

NF-κB activation is increasingly recognized as a key mechanism underlying malignancies that arise from chronic inflammatory processes [8,9]. Once activated, NF-κB regulates transcriptional programs that are crucial to immune defense and stress responses. Signaling outputs reported in glioma include a broad range of elevated cytokines and chemokines, such as interleukin (IL)-6, IL-1β, TNF-α, TGF-β, VEGF, CCL2/MCP-1, CXCL8/IL-8, and CXCL12, which drive proliferation, vascular activation and angiogenesis, BBB disruption, and the recruitment of immunosuppressive myeloid cells [10]. In parallel, the glioma microenvironment, characterized by hypoxia, necrosis, and immune infiltration, favors excessive production of reactive oxygen species (ROS) [11]. Redox regulation is a pivotal determinant of glioma progression, since both high and low ROS states can impair cellular survival [12]. As chemo- and radiotherapy partly rely on ROS-driven damage, these interventions may simultaneously eliminate susceptible tumor cells while enriching for resistant glioma stem cell (GSC) populations [13,14]. The hormetic model provides a useful framework to interpret this paradox, highlighting that insufficient ROS can sustain oncogenic adaptation, whereas excessive ROS can trigger cell death [15]. Consistent with this model, temozolomide (TMZ) sensitivity appears to depend on ROS accumulation and chaperone-mediated autophagy (CMA) activation, whereas resistance is associated with impairment of these processes; notably, this phenotype may be reversible through modulation of oxidative stress [16]. Importantly, effective translation of antioxidant and pro-oxidant therapies will depend on optimizing treatment timing, dosing, and combination strategies to overcome glioma adaptation while preserving normal brain tissue.

Cannabidiol (CBD) exhibits multiple pharmacological activities relevant to analgesic, anti-inflammatory, and anti-cancer properties [17]. Nonetheless, its clinical applicability remains limited due to low aqueous solubility (0.1 μg/mL), low oral bioavailability, and oxidation sensitivity, underscoring the need for improved formulation and stabilization strategies [18]. Celecoxib (CELE), classified as a cyclooxygenase (COX)-2-selective non-steroidal anti-inflammatory drug (NSAID), was originally indicated for pain and inflammatory symptoms in osteoarthritis and rheumatoid arthritis. However, beyond COX-mediated suppression of prostaglandin synthesis, CELE engages COX-2-independent pathways, thereby affecting cell cycle progression and survival [19,20]. Phase I/II trials exploring CELE-centered treatment strategies in recurrent malignant gliomas indicate an acceptable safety profile, yet limited permeability across the BBB may constrain brain and tumor concentrations following oral administration [21,22]. In line with the literature, liposomal delivery of NSAIDs can enhance drug solubility and therapeutic efficacy in aggressive tumors, as demonstrated for etoricoxib in breast cancer [23], indomethacin in non-small-cell lung cancer, and CELE and doxorubicin (DOXO) co-encapsulated in a melanoma model [24].

The improved efficacy observed with liposomal NSAID formulations reflects the intrinsic advantages of liposomal drug delivery. Liposomes have been developed as an effective strategy to improve therapeutic outcomes. Their biocompatible carrier structure protects encapsulated compounds and enhances targeted delivery to tumor cells. Rational design of liposomal lipid composition enables the modification of their physicochemical properties and biological behavior, including stability, BBB permeability, and cellular uptake. As membrane-mimetic nanocarriers, liposomes formulated from phosphatidylcholine-derived lipids are frequently used due to their low intrinsic toxicity and slightly negative surface charge. In turn, cationic lipid addition can enhance BBB transport by maximizing electrostatic adhesion to the endothelial barrier [25,26,27,28].

In this study, we developed two types of liposomal formulations containing CBD, CELE, and their combination to evaluate their anti-cancer potential in U-87 MG cells. The effects of the formulations were investigated with respect to cytotoxicity, apoptosis induction, cell cycle regulation, oxidative stress, and inflammatory signaling, with particular focus on the Nrf2, NF-κB, and Wnt/β-catenin pathways. To our knowledge, this study provides the first preliminary in vivo assessment of CBD + CELE-containing liposomal formulations targeting redox imbalance and inflammatory signaling in a glioma xenograft model. However, the present study does not provide direct structural confirmation of CBD and CELE co-localization within individual vesicles or pharmacokinetic confirmation of ratio-controlled co-delivery in tumor tissue.

## 2. Results

### 2.1. Liposome Size, Polydispersity Index, Zeta Potential Measurement, and Encapsulation Efficiency

**Figure 1:**
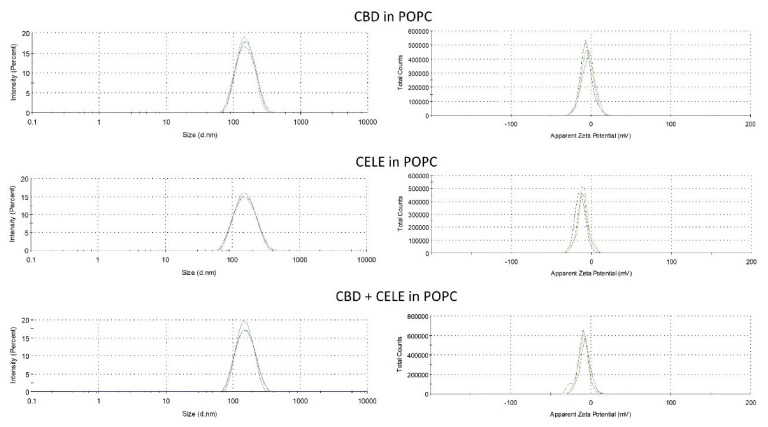
The size and zeta potential of the POPC liposomes (CBD-, CELE-, and CBD + CELE-loaded) were measured by dynamic light scattering (DLS).

The liposomal dispersions were characterized by hydrodynamic diameter (Z-average), polydispersity index (PDI), and zeta potential, as key indicators of colloidal homogeneity and electrostatic stability. Measurements were performed in triplicate immediately prior to each in vitro and in vivo experiment to ensure that the reported parameters reflected the formulations’ physicochemical state at the time of biological assessment. The results are summarized in Figure 1 for blank, CBD-, CELE-, and CBD + CELE-loaded single-lipid POPC-based liposomes (POPC lip.). Table 1 gives a brief overview of the physicochemical characteristics of in vitro-used POPC lip. and two-lipid DOTAP:POPC-based liposomes (DOTAP:POPC lip.), while Table 2 presents the parameters of in vivo-used liposomes. All preparations exhibited mean particle diameters below 157 nm and narrow size distributions, consistent with a homogeneous nanosuspension. Such uniformity is desirable for reproducible biodistribution and biological performance in subsequent in vivo studies, as nanoparticle size is a critical determinant of cellular internalization pathways and uptake efficiency. Particles in the sub-micrometer range (typically up to ~300 nm) may be internalized via endocytic mechanisms, including macropinocytosis, depending on cell type and nanoparticle surface properties [29,30].

Zeta potential represents the electrokinetic potential at the slipping plane of the electrical double layer surrounding dispersed nanoparticles (the Stern and diffuse layers), and provides insight into surface charge characteristics that influence interparticle repulsion, aggregation propensity, and interactions with biological interfaces. In general, dispersions with zeta potential magnitudes from >+30 mV to <+60 mV are considered electrostatically stabilized due to sufficient repulsive forces between particles [31]. In the present study, the zeta potential values of DOTAP:POPC liposomes ranged from +38.7 to +55.4 mV, confirming a distinctly cationic surface and supporting the colloidal stability of the liposomal suspension under the applied measurement conditions (Table 1 and Table 2).

Importantly, the formulations used for the in vitro and in vivo experiments should not be regarded as physicochemically identical preparations. Although they were based on the same principal lipid and drug components, they differed in final lipid/drug concentrations and preparation procedures. Therefore, the in vitro data characterize lower-concentration liposomal formulations, whereas the in vivo study should be interpreted as a preliminary evaluation of a related, higher-concentration formulation administered intratumorally, rather than as a direct assessment of the same physicochemical preparation.

From a biological perspective, an increase in surface charge may elevate both the toxicological risk of the carrier and its recognition and clearance by phagocytic cells. Although this limits their suitability for intravenous delivery, strongly cationic liposomes may be advantageous for local or intranasal administration, owing to their interactions with mucus and cellular surfaces [32,33]. Therefore, the selected zeta potential range represents a compromise between colloidal stability, enhanced membrane interaction, and acceptable biological tolerability under the applied experimental conditions.

Based on the linearity data obtained from the calibration curves, the amount of active substances encapsulated in the DOTAP:POPC lip. was calculated, followed by the determination of the encapsulation efficiency (EE) values with standard deviation (EE ± SD). The results are presented in Table 2.

### 2.2. Cytotoxicity and Apoptosis Induction of POPC Liposomes and DOTAP:POPC Liposomes in U-87 MG Glioma Cells

**Figure 2:**
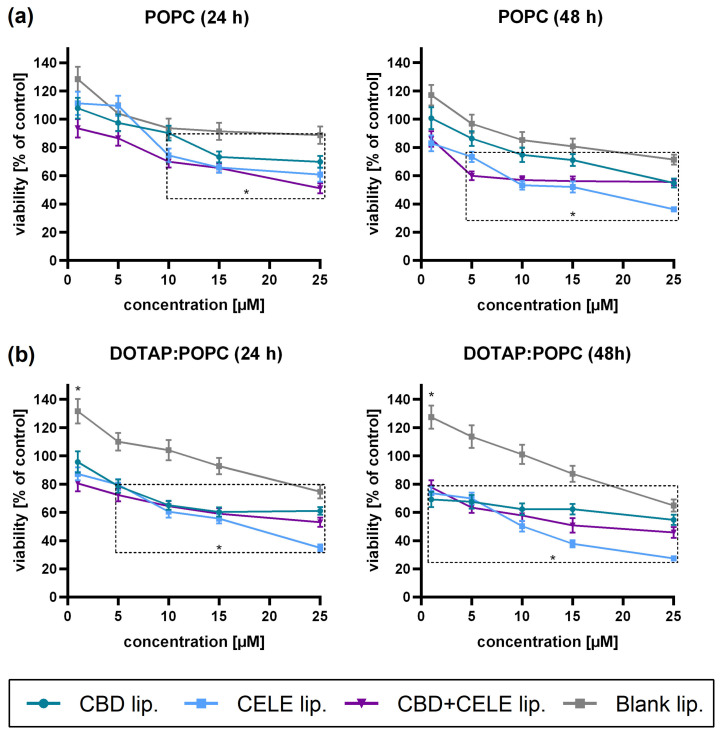
The results of the MTT assay following 24 h and 48 h of exposure of the U-87 MG cell line to POPC liposomes (**a**) and DOTAP:POPC liposomes (**b**) loaded with CBD, CELE, and their combinations, as well as blank liposomes. The square and asterisk (*) indicate statistically significant results compared to the control, considered as untreated cells with *p* < 0.05. Data are presented as mean values ± SEM from three independent experiments.

The inhibitory effect of blank, CBD-, CELE-, and CBD + CELE-loaded lip. on U-87 MG cell growth was initially assessed. Cancer cells were treated with graded concentrations of drug-containing formulations (1, 5, 10, 15, and 25  µM) for 24 and 48 h. Then, viability was measured using the MTT assay corresponding to mitochondrial metabolic activity (Figure 2). Compared to the non-treated cells, a reduction in cell viability to below 50% at 48 h was observed exclusively for CELE liposomes (POPC) within the tested range. In turn, the half-maximal inhibitory concentration (IC_50_) of CELE lip. (DOTAP:POPC) was 17.5 ± 1.4 µM for the 24 h treatment and 9.7 ± 1.8 µM for the 48 h treatment. Additionally, 48 h exposure to CBD + CELE formulations at 5 µM, representing the total concentration of both compounds, reduced U-87 MG cell viability by almost 40%.

**Figure 3:**
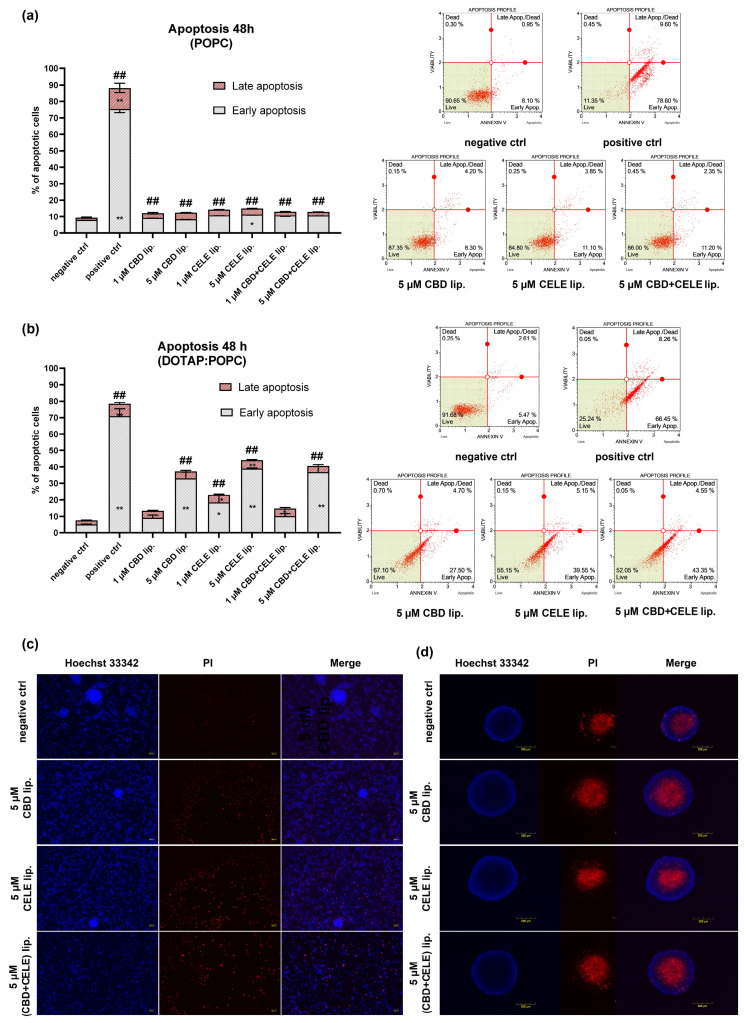
Apoptosis of U-87 MG cells treated with CBD, CELE, and their combinations loaded in POPC liposomes (**a**) and DOTAP:POPC liposomes (**b**) for 48 h. An asterisk (*) and double asterisk (**) indicate the values significantly different from the untreated control cells in early or late apoptosis phase with *p* < 0.05 and *p* < 0.01, respectively. Total apoptosis values significantly different from the untreated control are shown as double hashtag (##) with *p* < 0.01. Representative images of U-87 MG cells co-stained with Hoechst 33342 (blue) and propidium iodide (PI; red) after exposure to DOTAP:POPC liposomes for 48 h: cell monolayers (**c**) and spheroids (**d**). Scale bars, 100 µm and 200 µm, respectively.

Annexin V and 7-aminoactinomycin D (7-AAD) staining enable the distinction of viable, apoptotic, and late apoptotic/dead cells in flow cytometry, while Hoechst 33342 and propidium iodide (PI) are cellular dyes that allow for the observation of viable and dead cells microscopically. As illustrated in Figure 3, compared to POPC liposomes, DOTAP:POPC formulations exerted a greater pro-apoptotic impact on U-87 MG cells. POPC liposomes loaded with the same concentrations of the individual compounds or the mixture increased the percentage of total apoptotic cells, but did not achieve values above 20%. Interestingly, we observed the dominance of early phase apoptosis in cancer cells after 48 h of exposure to 5 µM DOTAP:POPC lip. loaded with CBD (33.1% of cells), CELE (39.0% of cells), or CBD + CELE (36.9% of cells). Additionally, we reported an increase in PI-positive dead cells in monolayer-grown U-87 MG cells upon treatment with CBD + CELE lip.

Using the spheroid-based 3D model, a central necrotic region was observed in both treated and untreated spheroids, with an increased area of red-stained apoptotic cells following exposure to the tested liposomes.

### 2.3. Encapsulated CBD + CELE and CELE in Cationic Liposomes Increased Cell Population in G_2_/M Phase

**Figure 4:**
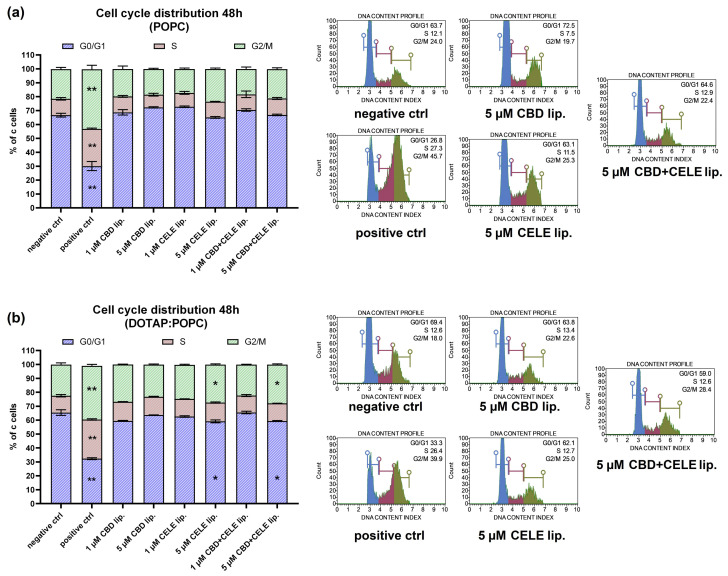
Cell cycle analysis of U-87 MG cell line treated with CBD, CELE, and their combinations loaded in POPC liposomes (**a**) and DOTAP:POPC liposomes (**b**) for 48 h using the Muse Cell Cycle Kit. Non-treated cells and DOXO (1 µM) served as negative and positive controls, respectively. Values are expressed as mean ± SEM from two independent experiments. An asterisk (*) and double asterisk (**) indicate values significantly different from the untreated control cells with *p* < 0.05 and *p* < 0.01, respectively. Representative histograms are also presented.

Measuring cell DNA content by PI staining allowed us to follow the distribution of cells in the G_0_/G_1_, S, and G_2_/M phases. In this way, we could observe cell cycle arrest in a single phase in response to the treatment. As shown in Figure 4, after exposure of U-87 MG to both types of liposomes loaded with CBD, CELE, and CBD + CELE, significant changes were observed only after treatment with CELE and CBD + CELE liposomes. It was observed that the percentage of U-87 MG cells in G_2_/M was significantly increased at 5 μM of cationic liposomes loaded with CELE and the combination, resulting in G_2_/M cell cycle phase arrest. Simultaneously, the population of cells in the G_0_/G_1_ phase significantly decreased to 59% in CELE- and combination-treated cells. Similarly, DOXO increased G_2_/M checkpoint engagement in glioma cells, consistent with its multimodal mechanism involving DNA damage, ROS production, apoptosis, senescence, autophagy, ferroptosis, and pyroptosis induction, as well as its immunomodulatory role [34].

### 2.4. Oxidative-Stress-Promoting Properties of Liposome-Loaded CBD + CELE After 48 h of Exposure

**Figure 5:**
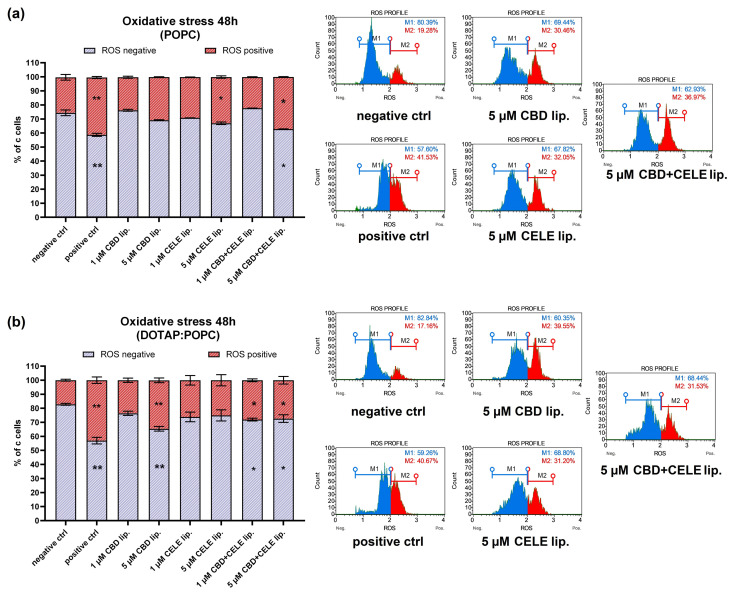
ROS profile was obtained in U-87 MG cell line treated with CBD, CELE, and their combinations loaded in POPC liposomes (**a**) and DOTAP:POPC liposomes (**b**) for 48 h using the Muse^®^ Oxidative Stress Kit. Non-treated cells and DOXO (1 µM) served as negative and positive controls of the assay, respectively. ROS (+) and ROS (−) indicate cells with detected and undetected superoxide radicals, respectively. Values are expressed as mean ± SEM from two independent experiments. An asterisk (*) and double asterisk (**) indicate values significantly different from the untreated control cells with *p* < 0.05 and *p* < 0.01, respectively. Representative histograms are also presented.

In malignancy, oxidative stress can amplify prosurvival and proliferation-associated signaling pathways, yet sufficiently strong redox perturbation may initiate a variety of cell death mechanisms, including ferroptosis, necroptosis, or pyroptosis [35]. Thus, we assessed whether liposome-loaded compounds can modulate intracellular redox homeostasis by measuring superoxide anions O_2_^•−^ levels in U-87 MG cells after a 48 h treatment using flow cytometry. Compared to the control group, the 5 μM CBD + CELE combination treatment increased U-87 MG cellular ROS levels approximately 1.8-fold (Figure 5). Importantly, the exposure to a low concentration of CBD + CELE cationic liposomes (1 μM) also caused a significant increase in the accumulation of intracellular superoxide radicals. Nevertheless, the strongest ROS increase was reported in CBD-treated U-87 MG cells, when loaded into DOTAP:POPC lip., achieving nearly 40% ROS-positive cells.

### 2.5. U-87 MG Cells Activate Antioxidant Nrf2 Signaling in Response to ROS Accumulation

**Figure 6:**
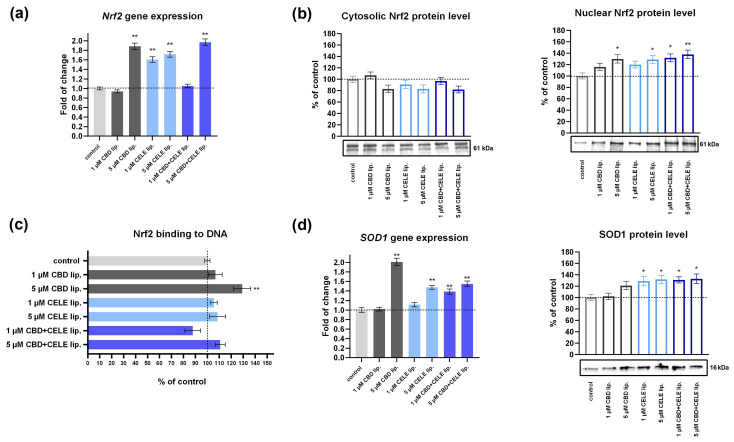
The effect of the DOTAP:POPC liposomes loaded with CBD, CELE, and their combinations on *Nrf2* mRNA expression (**a**); Nrf2 translocation from cytosol to nuclei (**b**); the Nrf2–DNA-binding capacity (**c**) and *SOD1* mRNA and SOD1 cytosolic protein expression (**d**) in U-87 MG cells after 48 h incubation. Data (mean ± SEM) are presented as fold changes relative to the control, which is defined as 100% for DNA-binding capacity and protein expression. A single asterisk (*) and double asterisk (**) denote statistical significance relative to the untreated control cells with *p* < 0.05 and *p* < 0.01, respectively.

A primary protective mechanism against excessive ROS is mediated by Nrf2 signaling, which promotes the expression of enzymatic detoxifiers such as superoxide dismutase 1 (SOD1), which catalyzes H_2_O_2_ degradation. Treatment with 5 μM CBD, CELE and their combination significantly increased mRNA expression of *Nrf2* in U-87 MG cells, resulting in 1.88-, 1.71-, and 1.96-fold increases, respectively (Figure 6a). The increased ROS level reported upon liposomal treatment triggered Nrf2 stabilization, as higher nuclear content of the Nrf2 protein was noted in treated cells (Figure 6b). In U-87 MG cells exposed to CBD, the nuclear Nrf2 level increased by 30% compared to the control, while the CBD + CELE combination exerted the strongest response in nuclear translocation by 38% (Figure 6b). Similarly, the DNA-binding ELISA results showed enhanced transcription factor binding with an antioxidant response element (ARE) by 29% and 11% in CBD- and combination-treated glioma cells (Figure 6c). Notably, 5 μM CBD, which produced the strongest increase in ROS, triggered enhanced expression of SOD1 at both the mRNA and protein levels by 2-fold and 21%, respectively (Figure 6d).

### 2.6. Strong Downregulation of NF-κB Activation After Treatment with CELE and CBD + CELE Liposomal Formulation

**Figure 7:**
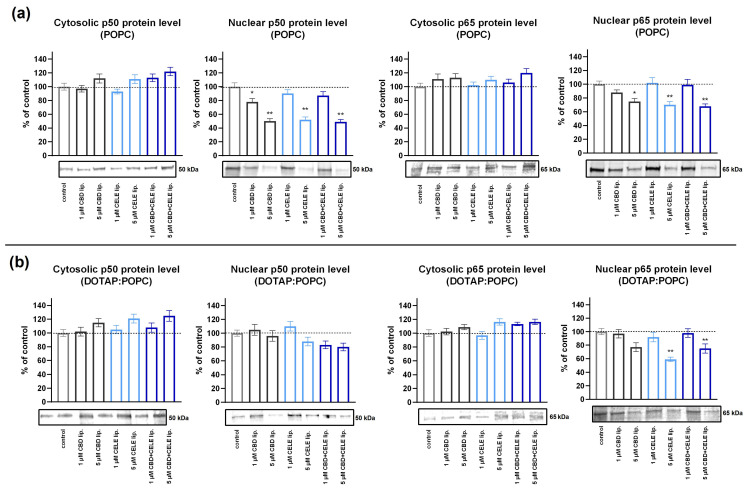
The effect of the POPC liposomes (**a**) and DOTAP:POPC liposomes (**b**) loaded with CBD, CELE, and their combinations on the translocation from cytosol to nuclei, NF-κB subunits p50 and p65 after 48 h incubation. Data (mean ± SEM) from three separate experiments are presented as fold changes relative to the control, which is defined as 100%. Bands are presented in the same sequence as the corresponding bars in the graph. A single asterisk (*) and a double asterisk (**) denote statistical significance relative to the untreated control cells, with *p* < 0.05 and *p* < 0.01, respectively.

Through interconnected signaling networks, oxidative stress and inflammation impact cell survival, tumor microenvironment, and treatment responses. To investigate the anti-inflammatory potential of the designed liposomes, we assessed their impact on NF-κB pathway activation and its targets, which correlate with glioma aggressiveness. Independent of the liposome type, nuclear translocation of the NF-κB p65 subunit was strongly reduced by CBD-, CELE- and CBD + CELE-loaded formulations (Figure 7). Western blotting demonstrated diminished p50 band intensities in nuclear extracts from U-87 MG cells exposed to 5 µM CBD + CELE liposomes, indicating attenuated NF-κB activation.

**Figure 8:**
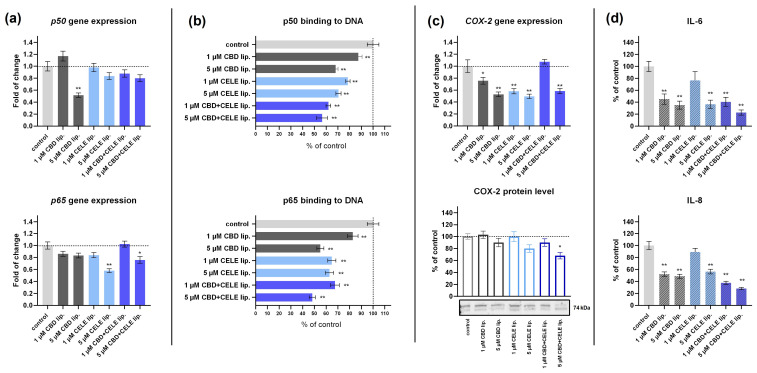
The effect of the DOTAP:POPC liposomes loaded with CBD, CELE, and their combinations on *p50* and *p65* gene expression (**a**); DNA-binding capacity of p50 and p65 (**b**) in U-87 MG cells after 48 h incubation. Panel (**c**) presents the *COX-2* gene expression and COX-2 protein levels in U-87 MG cytosolic fraction. Results of the multiplex MAGPIX immunoassay present IL-6 and IL-8 levels in U-87 MG cell lysates after 48 h of treatment (**d**). Data (mean ± SEM) from at least two separate experiments are presented as a fold of the control, which is defined as 100% for DNA-binding capacity and protein expression. A single asterisk (*) and double asterisk (**) denote statistical significance relative to the untreated control cells with *p* < 0.05 and *p* < 0.01, respectively.

Also, qPCR results showed a decrease in *p50* gene expression after liposomal CBD treatment. In contrast, significant changes in the *p65* gene encoding the other NF-κB subunit were observed after exposure to CELE and the combination (Figure 8a). Nevertheless, in all cases, we noted a decrease in p65 translocation from the cytosol to the nucleus, which was associated with reduced DNA-binding affinity of the NF-κB subunits. Similarly, after 48 h of exposure to the tested formulations, *COX-2* gene expression was reduced by half in single-loaded liposomes at 5 μM. The combination treatment suppressed *COX-2* gene expression, and we also reported marked changes in COX-2 protein levels after 48 h of exposure, with a 32% drop. Conversely, multiplex analysis of interleukins IL-6 and IL-8 showed a marked decline in cytokine levels, with the strongest suppression observed upon exposure to the 5 μM liposome-encapsulated combination by 77.4% and 72.0% compared with the non-treated control, respectively. Our findings suggest that the CBD + CELE liposomal combination may represent a promising strategy for suppressing inflammatory responses in glioma cells.

### 2.7. CBD-Loaded and Combination-Loaded Liposomes Reduced the Proliferation in U-87 MG Cells

**Figure 9:**
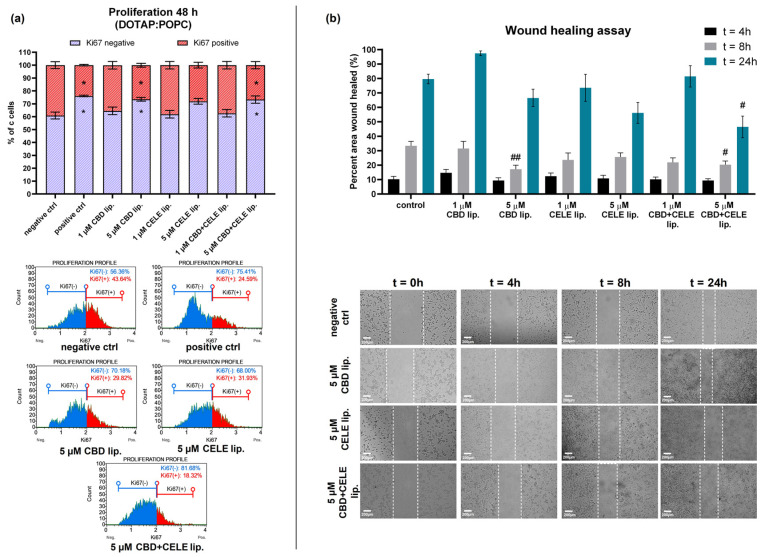
Proliferation marker Ki67 profile (**a**) was assessed in U-87 MG cell line treated with CBD, CELE, and their combinations loaded in DOTAP:POPC liposomes for 48 h using the Muse^®^ Ki67 Proliferation Kit. Non-treated cells and topotecan (1 µM) served as negative and positive controls, respectively. An asterisk (*) indicates values significantly different from the untreated control cells with *p* < 0.05. Representative histograms are also presented. The inhibition of the migratory potential of U-87 MG cells (**b**) as a result of treatment with CBD, CELE and their combinations loaded in DOTAP:POPC liposomes for 48 h was demonstrated using a wound-healing assay performed after 4, 8, and 24 h. Representative images of the wound-healing assay for control cells and cells treated with drug combinations are also presented (scale bar 200 µm). Data were pooled from two independent experiments with four measurements per assay. The results at each time point were statistically evaluated relative to the non-treated control. Single hashtag (#) and double hashtag (##) indicate values significantly different from the non-treated control at the same time point with *p* < 0.05 and *p* < 0.01, respectively.

High aggressiveness and infiltrative growth of glioma arise from the cells’ high proliferation index, invasive status, and self-renewal properties [36]. Ki67, a commonly used marker of proliferation, localizes to the nucleus in all cycling phases (G_1_, S, G_2_, and M), whereas it is not detected in cells in the G_0_ resting state. In our study, the liposome-loaded combination of CBD with CELE, as well as CBD alone, significantly decreased Ki67 expression (Figure 9a). Given that U-87 MG exhibits one of the highest proliferation rates, the observed modulation of this parameter is a promising finding. Next, a wound-healing assay was used to evaluate the effects of CBD, CELE, and their co-treatment on U-87 MG cell migration. As shown in Figure 9b, nearly total wound closure was achieved after 24 h in control cells and at a lower liposome concentration (1 µM). Treatment-caused migratory inhibition was first reported after 8 h for 5 µM CBD and the CBD + CELE combination. In this case, the wound healed to 33.4% in control cells, while exposure to CBD and CBD + CELE resulted in 17.1% and 20.3% healed area, respectively. Nevertheless, after 24 h, the inhibitory effect was sustained at 5 µM CBD + CELE exposure, with 46.5% wound closure, compared to 80% in the control.

**Figure 10:**
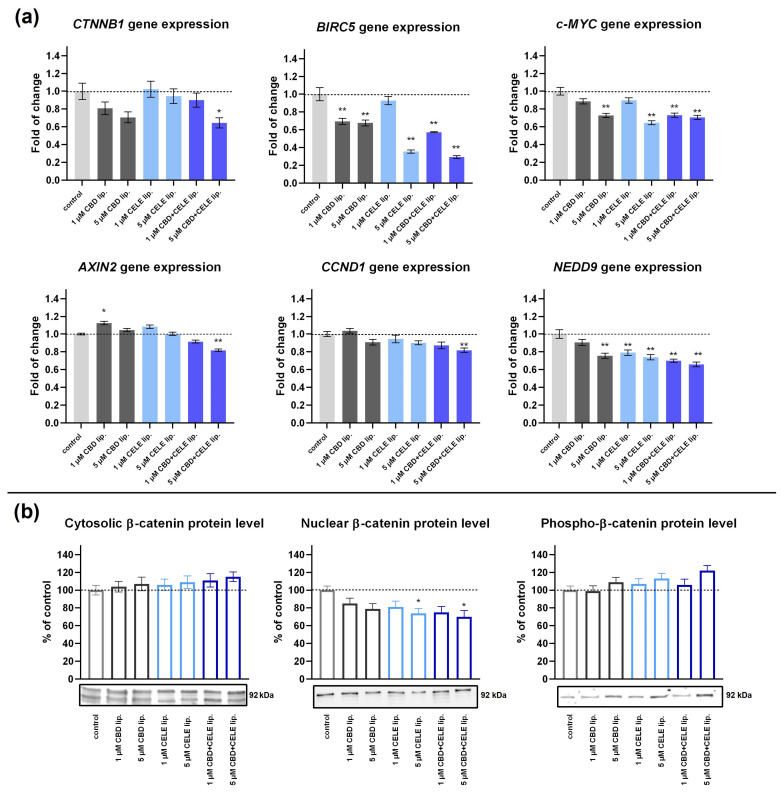
The effect of the DOTAP:POPC liposomes loaded with CBD, CELE, and their combinations on the Wnt/β-catenin target genes *CTNNB1*, *BIRC5*, *c-MYC*, *AXIN2*, *CCND1*, and *NEDD9* mRNA expression (**a**) and the β-catenin translocation from cytosol to nuclei and phospho-β-catenin level in cytosol (**b**) in U-87 MG cells after 48 h incubation. Data (mean ± SEM) from at least two separate experiments are presented as fold changes relative to the control, which is defined as 100% for protein expression. Representative immunoblots are shown for the analysis of β-catenin protein levels in the cytosolic and nuclear fractions, and of cytosolic phospho-β-catenin protein levels. A single asterisk (*) and double asterisk (**) denote statistical significance relative to the untreated control cells with *p* < 0.05 and *p* < 0.01, respectively.

Wnt signaling has been recognized as essential for neurodevelopment, controlling neurogenesis and maintaining neuronal system function. Notably, its misregulation has broad oncogenic consequences, including enhancing tumor aggressiveness and invasion, as well as maintaining stem-like cells with high self-renewal potential [37]. Higher-grade gliomas have been shown to exhibit increased β-catenin protein expression compared with lower-grade gliomas, consistent with their proposed role in proliferation and tumor growth [38]. The liposomal combination CBD + CELE treatment in U-87 MG cells significantly altered the target gene expression in the Wnt pathway. We reported a decrease in *CTNNB1* (0.36 vs. control), *BIRC5* (0.29 vs. control), *c-MYC* (0.70 vs. control), *AXIN2* (0.82 vs. control), *CCND1* (0.81 vs. control), and *NEDD9* (0.65 vs. control) gene expression (Figure 10a). The analysis of β-catenin protein expression in the cytosol and nuclei showed limited translocation of this central Wnt pathway mediator. The level of nuclear β-catenin after combination exposure decreased by 30% (Figure 10b). Similarly, CBD and CELE lip. treatment reduced the active β-catenin content in the nucleus by 21% and 26%, respectively. Simultaneously, cytosolic phosphorylated β-catenin indicated reduced β-catenin activation compared with control cells.

### 2.8. Combined CBD + CELE Liposomal Treatment Shows a Trend Toward Reducing Tumor Progression In Vivo

**Figure 11:**
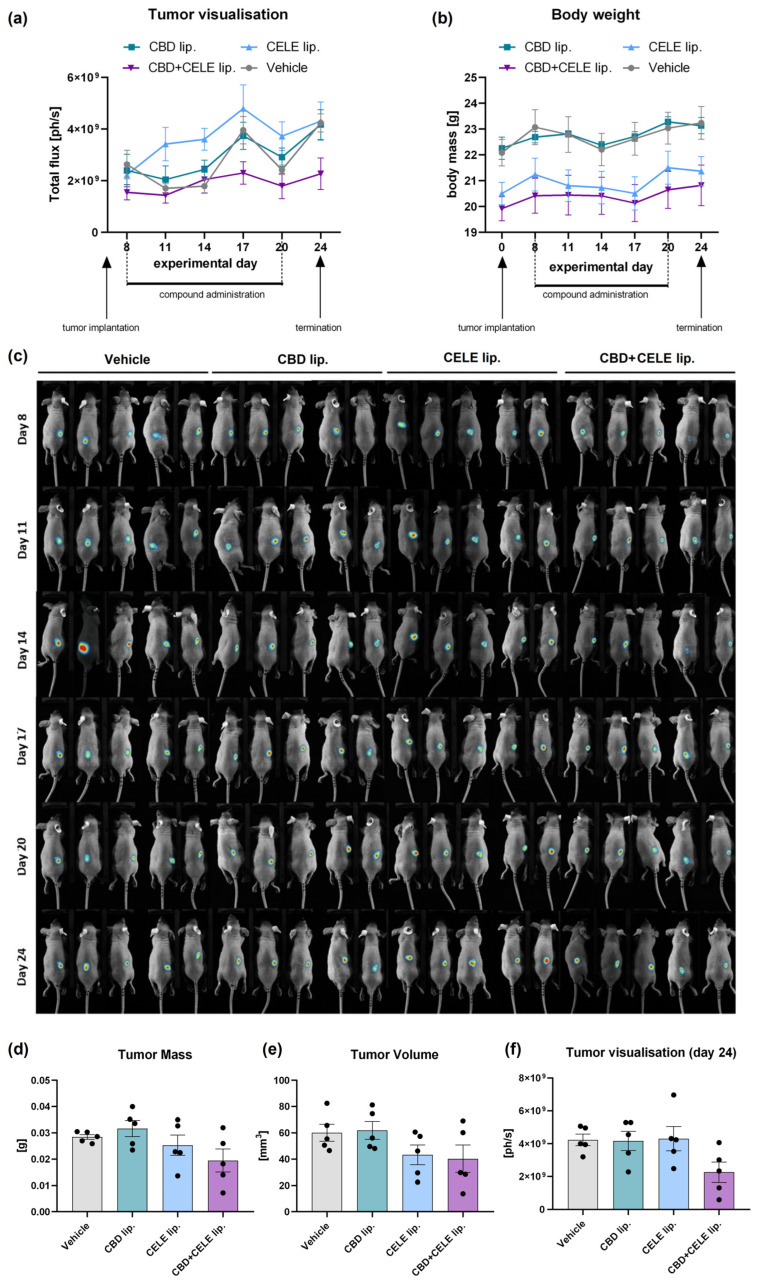
Tumor bioluminescence over time, represented as total flux (**a**) and body mass changes (**b**) in tumor-bearing BALB/c nude mice during the experiment. Animals received the control vehicle, CBD-loaded liposomes, CELE-loaded liposomes, and combination-loaded liposomes on days 8, 11, 14, 17, and 20 after tumor cell implantation. Bioluminescence imaging (**c**) was performed on days 8, 11, 14, 17, 20, and 24. Tumor mass (**d**), tumor volume (**e**), and tumor luminosity (**f**) were measured at the end of the experiment (day 24). In each of the 4 groups, 5 animals were analyzed. The data shown represents the mean ± SEM.

Tumor progression was monitored by measuring total bioluminescent flux over time (Figure 11a). Overall, tumor signal increased during the experimental period in most groups. The CELE-treated group showed the highest tumor-associated signal across multiple time points, peaking around day 17. The vehicle- and CBD lip.-treated groups showed comparable patterns, with moderate increases in signal intensity over time. In contrast, the combined CBD + CELE lip. treatment group consistently displayed the lowest bioluminescent signal throughout the study, suggesting reduced tumor progression compared with the other groups. To evaluate the therapeutic potential of CBD and CELE, tumor growth parameters, including mass (Figure 11d), volume (Figure 11e), and tumor luminosity (Figure 11f), were assessed across four experimental groups: vehicle control group, CBD lip.-treated group, CELE lip.-treated group, and a CBD + CELE combination lip.-treated group. Analysis of the terminal tumor mass revealed that monotherapy with either CBD or CELE lip. did not result in a significant reduction compared to the vehicle group. However, the combination therapy (CBD + CELE lip.) showed a slight downward trend in mean tumor mass, suggesting a potential additive inhibitory effect on tumor development. Consistent with the mass measurements, tumor volume was highest in the vehicle and CBD-only groups (averaging ~50–60 mm^3^). A noticeable decrease in tumor volume was observed in the group treated with the combination of CBD and CELE, indicating greater suppression of tumor expansion than with single-agent treatments.

Bioluminescence imaging was utilized to monitor tumor cell viability and progression in vivo (Figure 11c). By day 24, the CBD + CELE group exhibited the lowest signal intensity among all cohorts (Figure 11f). This reduction in photon flux suggests a trend toward a lower tumor-associated signal in the CBD + CELE-treated group compared with single-compound formulations.

To assess the systemic safety and physiological impact of the treatments, body mass (Figure 11b) and relative organ weights (Figure S1) were recorded. A significant reduction in total body mass was observed in the group receiving the combination of CBD and CELE compared to the vehicle control group (* *p* < 0.05). Mono-treatments with either CBD or CELE showed a slight but non-significant downward trend in body weight. Body weight remained relatively stable in all groups throughout the experiment. Mice in the vehicle and CBD groups showed a slight increase in body mass from approximately 22 g at baseline to about 23 g at day 24 (Figure 11b). In contrast, animals treated with CELE alone or in combination with CBD maintained lower body weights, ranging from approximately 20–21.5 g over the study period. Overall, no pronounced weight loss was observed during compound administration, indicating good tolerability of the treatments.

However, due to the limited group size and the absence of tissue drug-distribution data, these findings should be interpreted as preliminary evidence of antitumor-associated activity rather than proof of in vivo pharmacological synergy. Taken together, the in vivo data indicate a trend toward reduced tumor progression after intratumoral administration of the CBD + CELE-containing formulation. However, the study does not confirm the maintenance of the CBD:CELE ratio in tumor tissue or ratio-controlled co-delivery.

**Figure 12:**
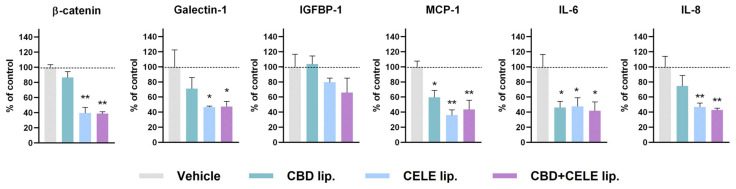
Effect of control vehicle (blank liposomes) and liposomes loaded with CBD, CELE, and CBD + CELE combination on glioma aggressiveness-related proteins, β-catenin, galectin-1, IGFBP-1, MCP-1, IL-6, and IL-8, measured by bead-based multiplex immunoassay in tumor homogenates derived from the in vivo study. Results are shown in comparison to vehicle control. The values are shown as the mean ± SEM calculated from at least three tumor samples. Significance of changes was determined by one-way ANOVA, post hoc Dunnett’s test (* *p* < 0.05, ** *p* < 0.01).

To further investigate the anti-inflammatory and invasiveness inhibition potential of liposomal treatment, we analyzed the level of glioma aggressiveness-related proteins, β-catenin, MCP-1, galectin-1, IGFBP-1, IL-6, and IL-8, in tumor homogenates. We observed a strong impact of CBD + CELE treatment on all tested proteins, except IGFBP-1, with a decreasing trend (Figure 12). The drop in expression was more than 50% compared to the control. For the tested parameters, no synergistic or additive effects were observed, as CELE lip. produced similar changes. In mice treated with CBD lip., MCP-1 expression decreased by 41% and IL-6 by 54%.

## 3. Discussion

This study was designed as a proof-of-concept evaluation of liposomal formulations containing CBD and/or CELE. The data indicate that formulations prepared with both compounds modulate redox balance, inflammatory signaling, and selected oncogenic pathways in U-87 MG cells, and show preliminary antitumor-associated activity in a subcutaneous xenograft model. However, because the study did not include single-vesicle structural confirmation, release-kinetics analysis, or LC-MS/MS-based tissue quantification of CBD and CELE, the findings should be interpreted as evidence of coordinated biological effects of CBD + CELE-containing liposomal preparations rather than as definitive evidence of ratio-controlled co-delivery or in vivo pharmacological synergy.

A key experimental observation was the enhanced biological activity of the CBD + CELE combination compared with that of single agents, consistent with our prior demonstration of synergism [39]. Mechanistically, this synergy appears to arise from convergent disruption of interconnected signaling axes, particularly Nrf2, NF-κB, and Wnt/β-catenin. While CBD primarily induces oxidative stress and redox imbalance, CELE simultaneously suppresses COX-2-driven inflammatory signaling and β-catenin activation.

Although the combination formulation did not consistently produce the strongest modulation of every individual molecular marker, the overall antitumor response likely reflects the simultaneous influence on several interconnected pathways, including oxidative stress, apoptosis, inflammatory signaling, proliferation, and migration. Therefore, the therapeutic activity of the CBD + CELE liposomal system should be interpreted as a multi-target biological effect rather than the consequence of the modulation of a single signaling pathway.

Importantly, these multi-target effects are closely linked to the properties of the delivery system. From a formulation perspective, both extrusion- and sonication-prepared liposomes achieved nanoscale size (<160 nm), which is relevant for tumor penetration and cellular uptake [40]. The lower PDI obtained by extrusion supports more predictable biological interactions, likely contributing to the consistency observed in vitro. However, partial loss of the drug during membrane extrusion cannot be excluded [41,42]. Thus, for the in vivo studies that required larger formulation volumes prior to administration, sonication was adopted as a more practical preparation method. Although its reproducibility is generally lower than that of extrusion, sonication still ensured an adequately homogeneous particle size distribution, while the molecular and nanoscale organization of the bilayer is expected to be largely independent of the preparation method [41]. Still, we consider the use of two different preparation procedures to be a limitation of the study. Also, although CBD and CELE were co-dissolved with lipid components in the same organic phase before film hydration, this preparation procedure alone does not prove that both compounds are co-localized within the same individual vesicles. Therefore, future studies should include dedicated structural and biophysical analyses, such as FTIR spectroscopy, differential scanning calorimetry, FRET-based approaches, or other suitable methods, to verify the distribution of both compounds within the liposomal population.

Beyond preparation-related considerations, liposome composition also critically influences their biological behavior. The formulation strategy involved initially developing a neutral reference POPC-based liposomal formulation, while the inclusion of DOTAP introduces a strong mechanistic component related to cellular uptake. The enhanced biological activity observed with DOTAP-containing formulations can be attributed to electrostatic interactions with negatively charged cell membranes, which promote membrane fusion and adsorption-mediated endocytosis [43]. In the context of brain tumors, this electrostatic component has been discussed as a contributor to adsorption-mediated endocytosis at the BBB and at the blood–brain tumor barrier (BBTB), potentially improving carrier uptake compared with neutral formulations. However, excessive cationic charge may also increase nonspecific protein adsorption, complement activation, and systemic clearance, necessitating careful optimization (e.g., PEGylation and/or ligand-mediated targeting) [44]. Notably, DOTAP-containing systems have been investigated in orthotopic glioblastoma models using PEGylated cationic liposomes (e.g., DOTAP/cholesterol/DSPE-PEG2000) further functionalized with targeting ligands such as transferrin, leveraging transferrin receptor (TfR) expression on both brain endothelial cells and glioma cells to support BBB/BBTB transport and tumor targeting [45,46]. Because in this study the formulations were administered intratumorally rather than intravenously, prolonged systemic circulation was not an intended formulation attribute. However, if systemic delivery or orthotopic glioma treatment is pursued in future studies, PEGylation or alternative surface-modification strategies should be evaluated together with pharmacokinetic, biodistribution, and toxicity analyses. In our work, DOTAP-based liposomal platforms were employed to enhance cellular interactions and support the biological evaluation of CBD-, CELE-, and CBD + CELE-containing formulations, underscoring the continued relevance of DOTAP as a cationic component in brain-tumor-oriented nanoformulation development [27,47]. The selection of the DOTAP:POPC 2:8 molar ratio was based on our earlier experiments [48,49] which identified this composition as effective for an advantageous balance between high drug content and efficient encapsulation. Nevertheless, encapsulation of substantially higher drug amounts for tumor-targeted administration in mice required scaling up the formulation, and the molar composition of the liposomes was modified accordingly.

While neutral and negatively charged liposomes are less effectively internalized by cells and display higher clearance rates, they are associated with a lower risk of adverse toxic effects [50,51]. The increased cytotoxicity of cationic liposomes can limit their applicability; therefore, we performed MTT viability assays in U-87 MG cells. The data showed that viability remained unaffected not only by empty POPC liposomes at low concentrations (1–10 µM) but also by liposomes formulated with the cationic lipid DOTAP. According to Cazzolla et al., storage temperature and time were identified as important determinants of DOTAP nanoparticle cytotoxicity, with freshly prepared and 1-week-stored liposomes exhibiting improved cellular compatibility relative to those stored for multiple weeks [52]. The observed changes in cell viability may reflect both the intrinsic activity of the encapsulated drugs and the biological contribution of the liposomal carrier, since strong interactions between cationic liposomes and cellular membranes can facilitate drug transport through membrane fusion, whereas at higher lipid concentrations they may also disturb membrane integrity and thereby enhance cytotoxicity [43,53]. We reported that early apoptosis accounted for the most prominent differences between loaded liposomes, with DOTAP:POPC producing the strongest effect. Additionally, we observed detectable effects on cell cycle regulation, with a trend towards G_2_/M phase arrest, which may be relevant for liposomal delivery in regimens combined with radiotherapy [54]. The moderate biological activity observed for blank cationic liposomes likely reflects the intrinsic membrane-interacting properties of DOTAP-containing formulations. Cationic lipids are known to enhance electrostatic interactions with negatively charged cellular membranes, potentially leading to mild membrane destabilization and increased cellular stress. Nevertheless, drug-loaded liposomes produced substantially stronger biological responses than blank formulations at equivalent lipid concentrations, indicating that the observed anti-glioma effects were predominantly associated with the encapsulated active compounds rather than the carrier itself.

A central mechanistic finding of our study is the dual role of oxidative stress and Nrf2 activation. Liposomal formulations of CBD, CELE, and CBD + CELE elicited a pro-oxidative cellular response, as previously described for both the free agents and those incorporated into carriers [39,55,56,57]. Interestingly, despite the stronger ROS induction after CBD treatment, the combination formulation did not proportionally enhance Nrf2 activation. This may indicate that CBD + CELE co-treatment affects redox homeostasis through partially distinct mechanisms, including simultaneous pro-oxidant and anti-inflammatory signaling modulation. Concurrently, an Nrf2-mediated cytoprotective response was triggered, evidenced by increased nuclear accumulation of Nrf2 and enhanced DNA-binding affinity. Interestingly, compared with CELE lip. alone, both CBD lip. and CBD + CELE lip. were associated with lower *Nrf2* expression levels at 1 µM. One possible explanation is that CELE has been reported to activate Nrf2 through a COX-2-independent AMPK/CREB/Nrf2 pathway, including AMPKα phosphorylation, CREB activation, and Nrf2 nuclear translocation [58]. By contrast, CBD seems to act as a pleiotropic regulator of oxidative stress and inflammation-related pathways with effects determined by concentration and cellular context [59]. Although Nrf2 activation may exert cytoprotective functions, transient Nrf2 induction in response to excessive ROS may also reflect an adaptive response to oxidative stress that ultimately exceeds the antioxidant capacity of glioma cells and contributes to cell death [60]. Alongside its canonical antioxidant role, elevated Nrf2 activity may drive autophagy, as Gianotti et al. demonstrated that low-dose CBD treatment increased autophagic flux, with increases in Beclin-1 and the LC3-II/LC3-I ratio [61]. Another study highlighted the role of Nrf2 in ferroptosis in TMZ-resistant cells, suggesting that high Nrf2 activity does not exclude overcoming resistance, providing alternative cell death mechanisms [62]. It should also be considered that long-term exposure to pro-oxidant agents may overwhelm compensatory repair pathways, enabling chronic oxidative stress to translate into lethal cellular effects. Therefore, we suggest that the observed Nrf2 activation may reflect a compensatory antioxidant response rather than a definitive effective cytoprotective outcome. Nevertheless, the enhancement of antioxidant signaling may raise concerns, underscoring the need for cautious conclusions and further investigation into redox adaptation and therapy resistance.

Prolonged oxidative stress may mechanistically prevent TNFα-induced activation of NF-κB, potentially involving not only proteasome inactivation but also impairment of components of the signaling cascade, such as decreased IκBα phosphorylation [63]. In our study, CELE lip. exerted a stronger suppressive effect on nuclear p65 levels than CBD + CELE lip., which was not initially expected. The non-additive effect of CBD and CELE in combination on NF-κB p65 subunit translocation may result from their convergence on the same signaling axis rather than from independent mechanisms. Since IKK activation precedes IκBα degradation and p65 release, CELE-mediated inhibition of IKK/Akt signaling could already strongly limit the pool of NF-κB available for nuclear translocation [64]. Under these conditions, CBD may not further suppress p65 translocation in an additive manner, particularly at low doses where its antioxidant and cytoprotective effects may preserve a residual NF-κB-dependent survival response [65].

In Ekiner et al.’s article, CBD was described as a modulator of redox status and inflammation, linking the oxidative stress response with Nrf2-NF-κB crosstalk [65]. Within the cytokine-rich glioma microenvironment (IL-1β, IL-6, TNF-α), NF-κB has been shown to act together with COX-2 and PGE2 to stimulate proliferation, invasion, angiogenesis, and immune escape [66,67,68]. We found that CBD and CELE, particularly when combined, downregulated NF-κB transcriptional activity, resulting in 44% and 52% reductions in p50 and p65, respectively. It has been demonstrated that CBD (1–10 μM) attenuated TNF-α and IL-1β production in LPS-stimulated murine microglial cells by suppressing NF-κB signaling [69]. In a mouse model of oral mucositis, CBD at 30 μM was reported to activate Nrf2, with these changes correlating with reduced NF-κB activity and lower TNF-α levels [70]. The interplay between Nrf2 and NF-κB provides an additional mechanistic layer. We observed significant reductions in NF-κB p50 and p65 activity following treatment, especially with the combination. This effect can be explained by competitive interactions for transcriptional coactivators (CBP/p300) and by ROS-mediated signaling changes that suppress NF-κB activation [71,72]. Also, CELE contributes to anti-inflammatory activity through the COX-2/PGE2 axis, which is a key source of pro-inflammatory prostaglandins that escalate inflammatory signaling in a positive feedback loop [73]. This interpretation is supported by the reduced levels of IL-6 and IL-8 observed after treatment, with the strongest suppression detected following exposure to the 5 μM liposome-encapsulated combination. Importantly, IL-6 reduction is particularly relevant given its role in maintaining glioma stemness and immune suppression, suggesting that the treatment may also modulate the tumor microenvironment in ways that favor antitumor immunity. The more pronounced reduction in nuclear p65 observed after CELE lip. treatment compared with the combination formulation may suggest that CELE is the dominant NF-κB-modulating component in this system. Nevertheless, the improved biological activity observed after combination treatment indicates that the therapeutic effect is unlikely to depend exclusively on NF-κB inhibition.

CELE’s contribution extends beyond COX-2 inhibition as CELE has been shown to modulate Wnt/β-catenin signaling in glioma [20], gastric [74], colon [75], and breast cancers [76]. The observed reduction in β-catenin activity indicates that CELE interferes with Wnt signaling, which is crucial for glioma proliferation and stem cell maintenance. Although the observed changes in β-catenin protein levels were moderate, the combination formulation significantly affected several Wnt/β-catenin target genes, including *CTNNB1*, *BIRC5*, *c-MYC*, and *NEDD9*. These findings suggest that functional modulation of Wnt-related transcriptional activity may occur even in the absence of major changes in total β-catenin protein abundance. CBD alone has limited direct effects on this pathway, but by inducing oxidative stress, it may sensitize cells to CELE-mediated inhibition. Therefore, the combination likely produces multi-node pathway disruption, targeting both upstream inflammatory signals and downstream proliferative drivers.

Combination approaches incorporating CELE have been pursued, but CELE has been noted to be ineffective as a chemosensitizer for DOXO in breast cancer [77]. Thus, CELE-based combinations should be designed with careful attention to tumor type, the co-administered drug, and the treatment regimen [77,78,79]. The potential for clinically significant cardiovascular and gastrointestinal adverse effects can limit CELE use in cancer therapy, yet combining agents may achieve therapeutic effects at reduced doses of individual drugs [80]. A combination of CBD and CELE was administered in a mouse model assessing its antidepressant and anxiolytic effects [81]. In turn, in subcutaneous HT-29 xenografts implanted in BALB/c nude mice, co-administration of CELE and 5-fluorouracil significantly limited tumor growth over time and decreased tumor mass at termination [82]. In androgen-insensitive PC-3 and androgen-sensitive LNCaP prostate cancer cells, CELE and genistein nanoliposomes produced a cell-line- and time-dependent reduction in viability accompanied by strong induction of cleaved caspase-3 combined with COX-2/GLUT1 targeting and redox stress induction [83]. The dose-responsive delay in tumor development under prophylactic dietary CELE was observed in a rat model, providing chemopreventive support for COXIB-based interventions [84]. Likewise, the latest report from Wang et al. provides an approach for CBD pretreatment as an immunomodulatory strategy, showing that 14 days of 10 mg CBD daily inhalation before intracranial tumor implantation in a murine model suppresses glioblastoma growth [85]. Zapata et al. synthesized CBD-loaded core–shell nanoliposomes that increased CBD solubility in simulated buccal and gastric environments and displayed a favorable selectivity profile, reducing viability of SW480 colon carcinoma cells while maintaining a non-toxic profile in HaCaT cells [86]. In glioma cell lines (U-87 MG and U-138 MG), acteoside and CBD combination-loaded liposomes promoted dose- and time-dependent apoptosis, evidenced by Bax induction and a reduction in the anti-apoptotic Bcl-xL [48]. However, in the study by Rao et al., CBD-loaded poloxamer 407 nanomicelles displayed improved biosafety and mitigated inflammatory cytokine release in vitro and in vivo [87].

Our in vivo study evaluated the effects of locally administered liposomal formulations on tumor growth and post-treatment modulation of oncogenic proteins. In the present study, different doses of CBD and CELE were intentionally used to account for the distinct pharmacological properties and effective concentration ranges of the two compounds. The dosing strategy was based on the prior literature but also considered the increased local drug availability, limiting systemic exposure compared to oral administration. Generally, oral CBD administration in mice has been reported at doses of ~1–100 mg/kg body weight, up to 150 mg/kg in some reports [88,89]. However, 10 mg/kg CBD has been applied peritumorally in a breast cancer model [90], and 5 mg/kg liposomal CBD has been given subcutaneously in dogs and goats for analgesia, collectively supporting a dose-sparing rationale for local liposomal dosing [91,92]. In this regard, published inflammatory mouse studies report using CELE at 40 mg/kg i.p., supporting our approach to minimize systemic toxicological risk [93]. A heterogeneous tumor microenvironment, including abnormal vascularization, uneven perfusion, elevated interstitial fluid pressure, dense extracellular matrix, hypoxia, and variable pH, may influence nanocarrier accumulation and local drug distribution [94]. Thus, further studies are needed to quantify CBD and CELE concentrations in tumor tissue and major organs over time to determine the relationship between administered dose, intratumoral exposure, and therapeutic efficacy.

Multiplex analysis showed that CBD and CBD + CELE treatments reduced galectin-1 abundance in tumor samples, a finding of potential relevance given the previous reports that U-87 MG cells are insensitive to OTX008, a galectin-1-targeting compound [95]. Also, we observed a decrease in tumor-associated cytokine levels (MCP-1, IL-6, and IL-8) upon CELE and CBD + CELE treatment, indicating an anti-inflammatory effect in glioma. In the Shono et al. study, CELE administered at 30 mg/kg/day downregulated MCP-1/CCL2 transcripts in a mouse glioma model, consistent with reduced CCL2 immunohistochemical staining [96]. Given the emerging role of IL-6 in shaping the glioblastoma immune microenvironment, reducing IL-6 supports the exploration of immunomodulatory strategies, as IL-6 neutralization enhances T-cell infiltration into glioblastoma tumors and improves survival in vivo [97]. In the β-catenin protein analysis, both single-loaded liposomal treatment and combination treatments do not show a significant difference in protein levels. However, in the in vivo tumor study, the combination therapy demonstrates improved efficacy.

Despite the promising results, this study’s limitations include the use of a single glioma cell line and a subcutaneous xenograft model, which may not fully recapitulate the complexity of human glioblastoma/astrocytoma or the BBB environment. The subcutaneous model may be regarded primarily as a screening platform for initial in vivo evaluation of antitumor activity, offering the advantage of reduced animal burden compared with intracranial implantation [98,99]. Therefore, further studies employing orthotopic models and additional high-grade glioma cell lines are warranted to validate the therapeutic potential of the proposed nanoformulations. It is important to note that although the liposomal strategy may significantly mitigate limitations related to solubility and simultaneous co-delivery, its translational potential remains challenging due to variability in intratumoral drug penetration. Accordingly, pharmacokinetic and brain biodistribution studies are required to determine whether the observed biological effects can be achieved at clinically relevant drug concentrations. Currently, clinical data are mostly based on case reports that demonstrate the reduction in tumor masses shown in tomography after CBD treatment of ovarian cancer patients [100], lung adenocarcinoma patients [101], and glioma patients. Regarding the latter group, GEINOCANN (phase Ib/II) assesses the feasibility of incorporating a tetrahydrocannabinol (THC):CBD product alongside TMZ-based therapy, and ARISTOCRAT (phase II) evaluates the clinical efficacy of THC:CBD with overall survival as the primary endpoint [102,103]. Our study provides evidence that not only the THC:CBD combination, but also the CBD:CELE combination warrants further investigation.

The present study has several limitations that should be considered when interpreting the findings. First, the in vitro and in vivo formulations were closely related in composition but not physicochemically identical, as they differed in final lipid/drug concentration and preparation procedure. Second, although CBD and CELE were incorporated into the same liposomal formulation, direct structural confirmation of their co-localization within individual vesicles was not obtained. Third, comprehensive characterization studies, including long-term storage stability, POPC degradation, drug retention, and release kinetics analyses, were beyond the scope of the current work. Fourth, quantitative LC-MS/MS measurements of CBD and CELE in tumor tissue, plasma, and major organs were not performed, limiting the assessment of intratumoral drug levels, biodistribution, pharmacokinetics, and systemic exposure. In addition, the subcutaneous xenograft model was used as an initial in vivo platform to evaluate biological activity; however, it does not fully recapitulate the complexity of glioma growth within the brain microenvironment. Consequently, the in vivo findings should be regarded as preliminary but encouraging and warrant further investigation in studies incorporating advanced formulation characterization, pharmacokinetic and biodistribution analyses, and orthotopic glioma models.

## 4. Materials and Methods

### 4.1. Chemicals

1-Palmitoyl-2-oleoyl-sn-glycero-3-phosphocholine (POPC) and 1,2-dioleoyl-3-trimethylammonium-propane (DOTAP) were purchased from Avanti Polar Lipids (Birmingham, AL, USA). Cannabidiol (CBD) was obtained from Medcolcanna Organics Inc. (Distrito Especial, Colombia), while celecoxib (CELE) was purchased from Pol-Aura (Zawroty, Poland).

### 4.2. Liposome Preparation

Liposomes were prepared according to a previously reported method using a lipid mixture of POPC and DOTAP [104]. Briefly, the lipids and active compounds were dissolved in organic solvents at the appropriate molar ratios. The solvents were removed under reduced pressure and then under high vacuum for 30 min to ensure complete removal of residual solvent. The resulting thin lipid film was subsequently hydrated with PBS (for in vitro studies) or sterile water (for in vivo studies).

#### 4.2.1. Formulations for In Vitro Studies

Small unilamellar vesicles (SUVs) and large unilamellar vesicles (LUVs) were prepared using an Avanti^®^ Polar Lipids Mini Extruder system (Merck KGaA, Darmstadt, Germany) equipped with a polycarbonate membrane (100 nm pore size). CBD, CELE, and their combinations were incorporated into the lipid phase at the following molar ratios:

− 0.1:13.5 (CBD or CELE:POPC) and 0.05:0.05:13.5 (CBD:CELE:POPC) for combined formulations;
− 0.1:8:2 (CBD or CELE:POPC:DOTAP) and 0.05:0.05:8:2 (CBD:CELE:POPC:DOTAP) for combined formulations.

The final liposome formulations contained 31.4 µg/mL of CBD and 38.1 µg/mL of CELE. In the combined formulations, the final concentrations of CBD + CELE were 15.7 + 19.1 µg/mL, respectively.

#### 4.2.2. Formulations for In Vivo Studies

Small unilamellar vesicles (SUVs) and large unilamellar vesicles (LUVs) were generated by sonication in an ultrasonic bath for 105–180 min at 22 °C. CBD, CELE, and their combinations were incorporated into the lipid phase at the following molar ratio: 8:80 (DOTAP:POPC). The final liposome formulations contained 1.33 mg/mL CBD, 5.33 mg/mL CELE, and a 1.33 + 5.33 mg/mL combination of both compounds.

The high-concentration formulation used for the in vivo experiment was not subsequently diluted and used for the in vitro assays. Therefore, the in vitro and in vivo experiments should be interpreted as complementary assessments of related liposomal systems rather than as direct characterization and efficacy testing of one identical formulation. In future studies, the high-concentration in vivo formulation should be prepared first and then diluted under controlled conditions for in vitro testing.

### 4.3. Characterization of Liposomes

#### 4.3.1. Liposome Size and Polydispersity Index Measurement

Dynamic light scattering (DLS) measurements were performed using the Zetasizer Nano ZS analyzer (Malvern Instruments, Malvern, UK) to determine particle size, reported as the hydrodynamic diameter (Z-average), and size distribution homogeneity, expressed as the polydispersity index (PDI). To achieve optimal particle concentration, 10 µL of liposome suspension was diluted with 10 mL of deionized water. Each formulation was measured in triplicate.

#### 4.3.2. Zeta Potential Measurement

Zeta potential was measured using electrophoretic light scattering (ELS) with a Zetasizer Nano ZS (Malvern Instruments, Malvern, UK). Samples were appropriately diluted with deionized water prior to measurement, and analyses were performed in triplicate. Measurements were conducted in U-shaped cuvettes equipped with gold electrodes The reported zeta potential values represent apparent zeta potentials calculated from electrophoretic mobility measurements using the Henry equation [105].

#### 4.3.3. HPLC Analysis and Encapsulation Efficiency

The drug concentration was determined using a direct method, i.e., by quantifying the active substance content in the formulation rather than measuring the non-encapsulated fraction in the dispersing medium. The obtained formulation containing encapsulated active compounds was dissolved in methanol to ensure complete solubilization of both the liposomal components (lipids and excipients) and the active substances. The resulting solution was subsequently analyzed using HPLC. The encapsulation efficiency (EE) was calculated based on a previously described method [49]. The actual amount of each compound entrapped in the liposomes was quantified using a validated HPLC method. The method was developed to enable the simultaneous determination of CBD and CELE in a single sample.

Liquid chromatography was performed on an RP-18 column under isocratic conditions. The mobile phase consisted of water and acetonitrile in a 15:85 (*v*/*v*) ratio, delivered at a flow rate of 0.8 mL/min. The HPLC system (Agilent 1220 Infinity LC, Agilent Technologies, Santa Clara, CA, USA) was equipped with a diode-array detector (DAD), a G1315C optical unit, an autosampler, and a column oven maintained at 25 ± 1 °C.

Detection wavelengths were set at 220 nm for CBD and 251 nm for CELE. The retention time was 6.4 min for CBD and 3.5 min for CELE, and the total analysis time was 10 min. A volume of 20 µL of each sample was injected, and all samples were analyzed in triplicate. Method validation included an assessment of selectivity, precision, linearity, range, and limits of detection and quantification. All used reagents were HPLC quality and delivered by Avantor Performance Materials (Gliwice, Poland).

### 4.4. Cell Culture

Human high-grade glioma (glioblastoma-astrocytoma) U-87 MG (HTB-14™) and U-87 MG-Luc2 (HTB-14-LUC2™) cells were obtained from the American Type Culture Collection (ATCC) and maintained in Eagle’s Minimum Essential Medium (EMEM) (Gibco, ThermoFisher Scientific, Waltham, MA, USA) supplemented with 10% fetal bovine serum (FBS). The 1% solution of penicillin–streptomycin (P/S) was added to EMEM to maintain U-87 MG cells, and blasticidin solution (at a final concentration of 8 µg/mL) was added to EMEM to maintain U-87 MG-Luc2 cells. Cell cultures were incubated at 37 °C in a humidified atmosphere containing 5% CO_2_.

### 4.5. Cell Viability MTT Assay

The U-87 MG cells were seeded at a density of 1 × 10^4^/well in a final volume of 200 μL in 96-well plates and incubated overnight. Next, the cells were exposed to liposomal formulations for 24 or 48 h at concentrations ranging from 1 to 25 μM, while negative controls were incubated in EMEM.

Following the incubation period, MTT (3-[4,5-dimethylthiazole-2-yl]-2,5-diphenyltetrazolium bromide) salt previously dissolved in medium (0.5 mg/mL) was added for 4 h. The precipitated formazan was solubilized in acidic isopropanol. Absorbance measurements were taken at 540 and 690 nm using an Infinite M200 microplate reader (TECAN, Grödig, Austria). All percentage values were calculated in relation to untreated control cells (100%). The experiment was performed in triplicate.

### 4.6. Hoechst 33342 and Propidium Iodide (PI) Staining

Approximately 5 × 10^3^ U-87 MG cells/well were seeded in 96-well plates and incubated for 24 h. Cell monolayers were treated with liposomal formulations at varying concentrations, while control cells were incubated in culture medium. After 48 h, cells were treated with PBS supplemented with fluorescent dyes: Hoechst 33342 and propidium iodide (PI) at final concentrations of 1 μg/mL and 2 μg/mL, respectively. Following 20 min of incubation in the dark at 37 °C, the solution was discarded, and the unbound dye was removed by PBS washing. Next, the plates were inserted into the CELENA^®^ X High Content Imaging System (Logos Biosystems, Annandale, VA, USA) for fluorescent microscopy.

Spheroids were established using U-shaped 96-well plates (Sarstedt AG & Co. KG, Nümbrecht, Germany). Viable U-87 MG cells (8  ×  10^3^/well) were seeded into each well in suspension in 100 μL EMEM supplemented with 10% FBS. The plates were incubated at 37 °C in a humidified atmosphere of 5% CO_2_. After 48 h, when spheroids were formed, the tested formulations were suspended in 100 μL of fresh medium and added to the spheroids. After 48 h, spheroids were treated with PBS containing the fluorescent dyes Hoechst 33342 and iodide (PI) at a final concentration of 5 μg/mL for each dye. Following 30 min of incubation in the dark at 37 °C, the plates were visualized as described above.

### 4.7. Flow Cytometry-Based Cell Analysis

#### 4.7.1. Apoptosis Analysis

U-87 MG cells were seeded in 12-well culture plates at a density of 5 × 10^4^ cells/well. Liposomal formulations containing either single compounds or combinations were then added to the culture medium at final concentrations of 1 or 5 μM, and cells were incubated for an additional 48 h. Doxorubicin (DOXO) at 1 µM was used as a positive control for apoptosis induction. Then, the collected cells were stained with annexin V and 7-aminoactinomycin D (7-AAD) solution for 20 min according to the Annexin V & Dead Cell Kit protocol (Merck KGaA, Darmstadt, Germany). Using the Muse^®^ Cell Analyzer (Merck KGaA, Darmstadt, Germany), flow cytometry was performed to evaluate differentiation between live, early apoptotic, late-apoptotic, and necrotic cells. The obtained data were processed with Muse^®^ 1.4 Software (Merck KGaA, Darmstadt, Germany).

#### 4.7.2. Cell Cycle Distribution Analysis

U-87 MG cells were seeded at 5 × 10^4^ cells/well in 12-well plates. The next day, cells were treated with liposomal formulations containing single or combined CBD and CELE at concentrations of 1 or 5 μM. Positive control for this assay was performed with DOXO (1 µM). After 48 h of incubation, cells were collected, fixed in 70% ice-cold ethanol, and stored at −20 °C overnight for subsequent procedures. Then, cells were carefully washed with PBS and incubated for 30 min with the reagent mixture prepared using the Muse^®^ Cell Cycle Kit (Merck KGaA, Darmstadt, Germany), and analyzed by flow cytometry using the Muse^®^ Cell Analyzer (Merck KGaA, Darmstadt, Germany). All measurements were processed by Muse^®^ Analysis Software version 1.4 (Merck KGaA, Darmstadt, Germany).

#### 4.7.3. Oxidative Stress Analysis

U-87 MG cells were seeded in 12-well plates (5 × 10^4^ cells/well) and cultured for 24 h before treatment with liposomal formulations (1 and 5 μM). DOXO-treated (1 µM) and untreated cells served as positive and negative controls, respectively. After 48 h of exposure, cells were harvested, rinsed with PBS, and centrifuged at 300× *g* for 5 min. The pellet was resuspended in Assay Buffer, after which 10 μL of the suspension was mixed with 190 μL of Muse^®^ Oxidative Stress working solution freshly prepared according to the Muse^®^ Oxidative Stress Kit protocol (Merck, Darmstadt, Germany). After 30 min incubation in the dark, the percentage of ROS-positive cells was quantified on the Muse^®^ Cell Analyzer (Merck KGaA, Darmstadt, Germany). Data were analyzed using Muse^®^ 1.4 Software (Merck KGaA, Darmstadt, Germany).

#### 4.7.4. Cell Proliferation Analysis

A total of 5 × 10^4^ cells/well were seeded in 12-well plates and incubated for 24 h to allow cell attachment. Then, cells were exposed to 1 and 5 μM liposomal formulations for 48 h. Topotecan (1 µM) was used as a positive control. Next, cells were fixed and permeabilized, then incubated with the staining reagents supplied in the Muse^®^ Ki67 Proliferation Kit (Merck KGaA, Darmstadt, Germany). Data acquisition was performed on the Muse^®^ Cell Analyzer, and analysis was conducted using Muse^®^ Analysis Software version 1.4 (Merck KGaA, Darmstadt, Germany).

### 4.8. Wound-Healing Assay

To assess the migration capacity of U-87 MG cells after exposure to the tested compounds, we seeded cells at 1.5 × 10^4^ cells per well in 12-well plates to reach ∼95% confluence after 24 h. Cell monolayers were scratched using a sterile pipette tip. Subsequently, wells were washed with PBS to remove cell debris and treated with 1 μM and 5 μM of the nanoformulations. To minimize the influence of proliferation on migration analysis, cells were exposed to relatively low concentrations of the tested formulations. The scratches were photographed under the Millicell^®^ DCI Digital Cell Imager (Merck, Darmstadt, Germany) at 10 magnifications at 0, 4 h, 8 h, and 24 h intervals. The average area of the wound was measured using ImageJ software version 1.8.0. The experiment was repeated 2 times with 4 wells per analyzed compound/s per assay. Data from multiple wells were pooled, and overall migration was determined by using the mean control premigration area as a reference and subtracting the final migration area for each condition.

### 4.9. Quantitative Real-Time PCR

U-87 MG cells were plated into 6-well plates at 3 × 10^5^ cells/well and treated with liposomal formulations at 1 and 5 μM for 48 h. RNA extraction was performed with the GeneMatrix Universal DNA/RNA/Protein Purification Kit (EurX, Gdańsk, Poland) according to the supplier’s protocol. RNA quantity and purity were determined using a NanoDrop^®^ spectrophotometer (ThermoFisher Scientific, Waltham, MA, USA). The Revert-Aid First Strand cDNA Synthesis Kit (Thermo Fisher Scientific, Waltham, MA, USA) was used for reverse transcription, and real-time PCR analyses were performed using the Maxima SYBR Green Kit (Thermo Fisher Scientific, Waltham, MA, USA) on the LightCycler 96 version 1.1.0.1320 (Roche, Basel, Switzerland). Experimental conditions were applied as described previously [55]. Relative gene expression was calculated using the 2^−ΔΔCt^ method, with TBP (TATA box-binding protein) and PBGD (porphobilinogen deaminase) used as reference genes for normalization. Primer sequences are presented in Table 3.

### 4.10. Transcription Factor Binding Assay

Nuclear fractions from liposome-exposed U-87 MG cells were isolated using the Nuclear/Cytosol Fractionation Kit (Abcam, Cambridge, UK) and analyzed for Nrf2 and NF-κB activation with TransAM™ ELISA kits (Active Motif, Carlsbad, CA, USA). Briefly, 2 μL of each nuclear fraction, diluted in lysis buffer to the recommended concentration, were added to wells coated with double-stranded oligonucleotides containing the specific consensus binding sequences for Nrf2/ARE or NF-κB subunits (p50, p65). The plates were incubated for 1 h at room temperature with mild agitation. Then, wells were washed extensively before sequential 1 h incubation with primary antibodies, followed by incubation with HRP-conjugated secondary antibodies applied for an additional hour. Colorimetric development was quantified at 450/655 nm using the Infinite M200 microplate reader (TECAN, Grödig, Austria).

### 4.11. Western Blot Analysis

U-87 MG cells (3 × 10^5^/well) were grown in 6-well plates for 24 h, treated with nanoformulations (1 and 5 μM) for an additional 48 h, and harvested for cytoplasmic and nuclear isolation using the Nuclear/Cytosol Fractionation Kit (Abcam, Cambridge, UK) following the manufacturer’s instructions. Total protein levels were measured by the Lowry method based on colorimetric analysis [106]. Samples containing 100 μg of nuclear (Nrf2, NF-κB p50, NF-κB p65, β-catenin) or cytosolic proteins (Nrf2, SOD1, NF-κB p50, NF-κB p65, COX-2, β-catenin, phospho-β-catenin) were subjected to electrophoresis on Mini-PROTEAN^®^ TGX Stain-Free™ Protein Gels (Bio-Rad Laboratories, Hercules, CA, USA) and transferred onto a nitrocellulose Immobilon P membrane (Sigma-Aldrich, St. Louis, MO, USA). To eliminate nonspecific binding, membranes were blocked for 2 h in skimmed milk, then incubated with primary antibodies overnight. After triple washing in DPBS, the membranes were incubated with secondary antibodies labeled with alkaline phosphatase (AP) or horseradish peroxidase (HRP). Immunoreactive signal intensities were quantified using a Bio-Rad ChemiDoc™ imaging system (Bio-Rad Laboratories, Hercules, CA, USA). Normalization of band intensities to total protein was achieved using stain-free imaging technology (Bio-Rad Laboratories, Hercules, CA, USA) [107]. Densitometric analysis was performed with ImageLab version 6.1.0 (Bio-Rad Laboratories, Hercules, CA, USA).

### 4.12. Cell Lysate Extraction

U-87 MG cells (2.5 × 10^4^ /well) were grown in 6-well plates for 24 h, treated with nanoformulations (1 and 5 μM) for an additional 48 h, and lysated using cell extraction buffer (Invitrogen, ThermoFisher Scientific, Waltham, MA, USA). Briefly, scraped cells were washed in ice-cold PBS, then centrifuged (at 600× *g* for 10 min at 4 °C) to remove the supernatant. The cell pellet was incubated on ice with 80 μL of cell extraction buffer, vortexing every 10 min. After centrifugation (at 13,000× *g* for 10 min at 4 °C), lysates were collected and stored at −80 °C.

### 4.13. In Vivo Experimental Protocols and Therapeutic Scheme

Homozygous male BALB/c nude mice (CAnN.Cg-Foxn1^nu^/Crl) (9 weeks old) were anesthetized with isoflurane and inoculated subcutaneously in the right flank with 1 × 10^6^ U-87 MG-Luc2 cells suspended in 200 µL of 50% MatriGel (Corning Life Science, Tewksbury, MA, USA) and DMEM culture medium (Gibco, ThermoFisher Scientific, Waltham, MA, USA) without FBS and antibiotics. In accordance with the principle of reduction, only the minimum number of animals (n = 20) required to achieve the study aims was included. After 8 days, male mice were randomly divided into 4 groups: (1) vehicle control group (n = 5); (2) CBD lip.-treated group (n = 5); (3) CELE lip.-treated group (n = 5); and (4) CBD + CELE lip.-treated group (n = 5). Blank DOTAP:POPC (in molar ratio 8:80) lip., CBD lip. (corresponding to 10 mg/kg b.w of CBD), CELE lip. (corresponding to 40 mg/kg b.w of CELE), and CBD + CELE lip. (corresponding to 10 and 40 mg/kg b.w of CBD and CELE, respectively) were administered intratumorally at days 8, 11, 14, 17, and 20 post-implantation. Group allocation was known at all stages of the experiment. Tumor growth was monitored using the bioluminescence method with Vilber Newton 7.0 equipment. The tumor volume and body weight of each mouse were measured during the whole experimental process. Tumor area (mm^2^) and volume (mm^3^) were calculated using the formulas: area = a × b, volume = 1/2 a × b^2^, where a = the longer side of the tumor and b = the shorter side of the tumor. At the end of the experiment, mice were terminated by isoflurane overdose.

During acclimatization and throughout the experiment, the animals were maintained in ventilated cages, under controlled environmental conditions: daily cycle of 12 h of light and 12 h of darkness, at a temperature of 22 °C ± 2 °C, and an air humidity of 55–60%. All groups’ animal handling and measurements were conducted at similar intervals and in comparable conditions. None of the animals were excluded from the experiment.

### 4.14. Homogenization of Tumor Samples

Each frozen tumor sample was placed in ceramic bead-containing tubes with 100 µL ice-cold Cell Lysis Buffer (Invitrogen, Thermo Fisher Scientific, Waltham, MA, USA). Homogenization was performed on a Precellys Evolution Touch homogenizer (Bertin, France) in three 20 s cycles, separated by 1 min cooling intervals. After centrifugation (at 16,000× *g* for 10 min at 4 °C), supernatants were collected and stored at −20 °C.

### 4.15. Bead-Based Immunoassay on the Luminex MAGPIX

A magnetic bead-based multiplex immunoassay was performed using the Luminex xMAP^®^ platform. The high-sensitivity magnetic bead panel PROCARTAPLEX—for analytes β-catenin, galectin-1, IGFBP-2, IL-6, IL-8 (CXCL8), and MCP-1 (CCL2) (Invitrogen, ThermoFisher Scientific, Waltham, MA, USA)—was used to quantify the parameters in lysates of treated U-87 MG cells and tumor homogenates from treated mice according to the manufacturer’s instructions. Briefly, cell lysates or tumor homogenates were diluted in the Universal Assay Buffer and transferred to a 96-well plate. A suspension of antibody-coupled magnetic beads was added to each well, followed by samples, and the plate was incubated for 2 h on a shaker, protected from light. After washing, the solution of Biotinylated Detection Antibody Mix was added and incubated for 30 min at room temperature with shaking. Subsequently, Streptavidin–PE (SA-PE) solution was added to generate the reporter signal and to prepare the plate for analysis on a MAGPIX^®^ instrument; the plate was then read in reading buffer for 5 min. The plate was inserted into the MAGPIX^®^ analyzer, which identifies each bead region and records reporter fluorescence. Raw mean fluorescence intensity (MFI) values were collected using Luminex XPONENT for MAGPIX software, version 4.2 (Luminex Corporation, Austin, TX, USA) and further processed in MILLIPLEX^®^ Analyst 5.1 (EMD Millipore, Burlington, MA, USA). For data presentation, MFI values were normalized to the untreated control (relative to control).

### 4.16. Statistical Analysis

The data were processed in GraphPad Prism 9.2.0 (GraphPad Software, San Diego, CA, USA). For in vitro experiments, one-way ANOVA with Dunnett’s post hoc test was used to determine significant differences; *p* < 0.05 was considered statistically significant.

For in vivo experiment results, a two-way ANOVA with Tukey’s correction was used to determine statistical significance for graphs depicting the distribution of body weight over time and tumor luminosity over time. One-way ANOVA with Holm–Sidak’s correction was used to determine statistical significance for graphs depicting body weight, tumor weight, tumor volume and surface area, and tumor luminosity on the last day of the experiment. For non-normal data, the Kruskal–Wallis test with Dunn’s correction was used. In addition, Student’s *t*-test was used for normally distributed data, and the Mann–Whitney U test was used for non-normally distributed data.

## 5. Conclusions

In summary, this study shows that liposomal formulations containing CBD and CELE, particularly DOTAP:POPC formulations prepared with both compounds, modulate apoptosis, cell cycle distribution, oxidative stress, Nrf2/NF-κB/Wnt-related pathways, and glioma-associated inflammatory mediators in U-87 MG-based models. In the subcutaneous xenograft model, intratumoral administration of the CBD + CELE-containing formulation was associated with a trend toward reduced tumor progression and modulation of selected tumor-associated proteins. These findings support further investigation of CBD + CELE-containing liposomal systems but do not establish physicochemical equivalence between the in vitro and in vivo preparations, single-vesicle co-localization of CBD and CELE, ratio-controlled co-delivery, or in vivo pharmacological synergy. Further studies should include controlled formulation-bridging experiments in which a high-concentration in vivo formulation is prepared and subsequently diluted under controlled conditions for in vitro testing, structural and biophysical characterization, stability and release-kinetics analyses, LC-MS/MS-based pharmacokinetic and biodistribution studies, toxicity assessment, and validation in orthotopic glioma models.

## Figures and Tables

**Table 1:** Physicochemical characteristics of liposomes for in vitro studies: particle size, polydispersity index (PDI), and zeta potential.

Formulation	Particle Size [nm]	PDI	Zeta Potential [mV]
blank liposomes (POPC lip.)	155.0	0.17	−17.9
CBD in POPC lip.	144.0	0.08	−4.6
CELE in POPC lip.	144.8	0.10	−12.0
CBD + CELE in POPC lip.	144.9	0.09	−9.2
blank liposomes (DOTAP:POPC lip.)	147.8	0.10	+38.7
CBD in DOTAP:POPC lip.	147.0	0.17	+42.6
CELE in DOTAP:POPC lip.	156.9	0.17	+46.4
CBD + CELE in DOTAP:POPC lip.	151.5	0.18	+45.4

**Table 2:** Physicochemical characteristics of liposomes for in vivo studies: particle size, polydispersity index (PDI), zeta potential, and encapsulation efficiency (EE).

Formulation	Particle Size [nm]	PDI	Zeta Potential [mV]	EE (±SD) [%]
blank liposomes (DOTAP:POPC lip.)	81.2	0.38	+52.7	-
CBD in DOTAP:POPC lip.	109.2	0.27	+51.8	99 (±1)
CELE in DOTAP:POPC lip.	113.4	0.40	+55.4	99 (±2)
CBD + CELE in DOTAP:POPC lip.	98.8	0.26	+49.4	98 (±1) + 100 (±2)

**Table 3:** The list of primers used in real-time PCR.

	Forward Primer	Reverse Primer	Accessible Number
*TBP*	5′-GGCACCACTCCACTGTATC-3’	5′-GGGATTATATTCGGCGTTTCG-3’	NM_003194.5
*PBGD*	5′-TCAGATAGCATACAAGAGACC-3′	5′-TGGAATGTTACGAGCAGTG-3′	NM_000190.4
*Nrf2*	5′-ATTGCTACTAATCAGGCTCAG-3′	5′-GTTTGGCTTCTGGACTTGG-3′	NM_006164.5
*SOD1*	5′-CGACAGAAGGAAAGTAATG-3	5′-TGGATAGAGGATTAAAGTGAGG-3′	NM_000454.5
*NF-κB p50*	5′-ATCATCCACCTTCATTCTCAA-3′	5′-AATCCTCCACCACATCTTCC-3′	NM_003998.5
*NF-κB p65*	5′-CGCCTGTCCTTTCTCATC-3′	5′-ACCTCAATGTCCTCTTTCTG-3′	NM_021975.4
*COX-2*	5′ CCTGTGCCTGATGATTGC-3‘	5′-CAGCCCGTTGGTGAAAGC-3′	NM_000963.4
*CTNNB1*	5′-GGTGACAGGGAAGACATC-3′	5′-GACAAAGGGCAAGATTTCG-3′	NM_001904.4
*BIRC5*	5′-GGACCACCGCATCTCTAC-3′	5′-CCTTGAAGCAGAAGAAACAC-3′	NM_001168.3
*AXIN2*	5′-TAGGTTCTGGCTATGTCTTTGC-3′	5′-GCCTTCACACTGCGATGC-3′	NM_004655.4
*c-MYC*	5′-TTACAACACCCGAGCAAG-3′	5′-AATCCAGCGTCTAAGCAG-3′	NM_002467.6
*CCND1*	5′-CCCTCGGTGTCCTACTTC-3′	5′-TCCTCGCACTTCTGTTCC-3′	NM_053056.3
*NEDD9*	5′-GAACAAGAGGTATATCAGGTG-3′	5′-TTGAGTGGTATGAGAAGGAG-3′	NM_006403.4

## Data Availability

The original contributions presented in this study are included in the article/Supplementary Materials. Further inquiries can be directed to the corresponding author.

## Supplementary Materials

The following supporting information can be downloaded at: https://www.mdpi.com/article/10.3390/ijms27146220/s1.
