# Supplementary material for: Cannabidiol- and Celecoxib-Loaded Liposomes as a Strategy to Modulate Redox and Inflammatory Signaling in High-Grade Glioma: A Preliminary In Vivo Study
Source: Int J Mol Sci. 2026 Jul 12;27(14):6220. doi: 10.3390/ijms27146220 (PMC13410183; doi:10.3390/ijms27146220)
Supplement: Supplementary file 1 [file ijms-27-06220-s001.zip › Supplementary Figure S1.pdf]

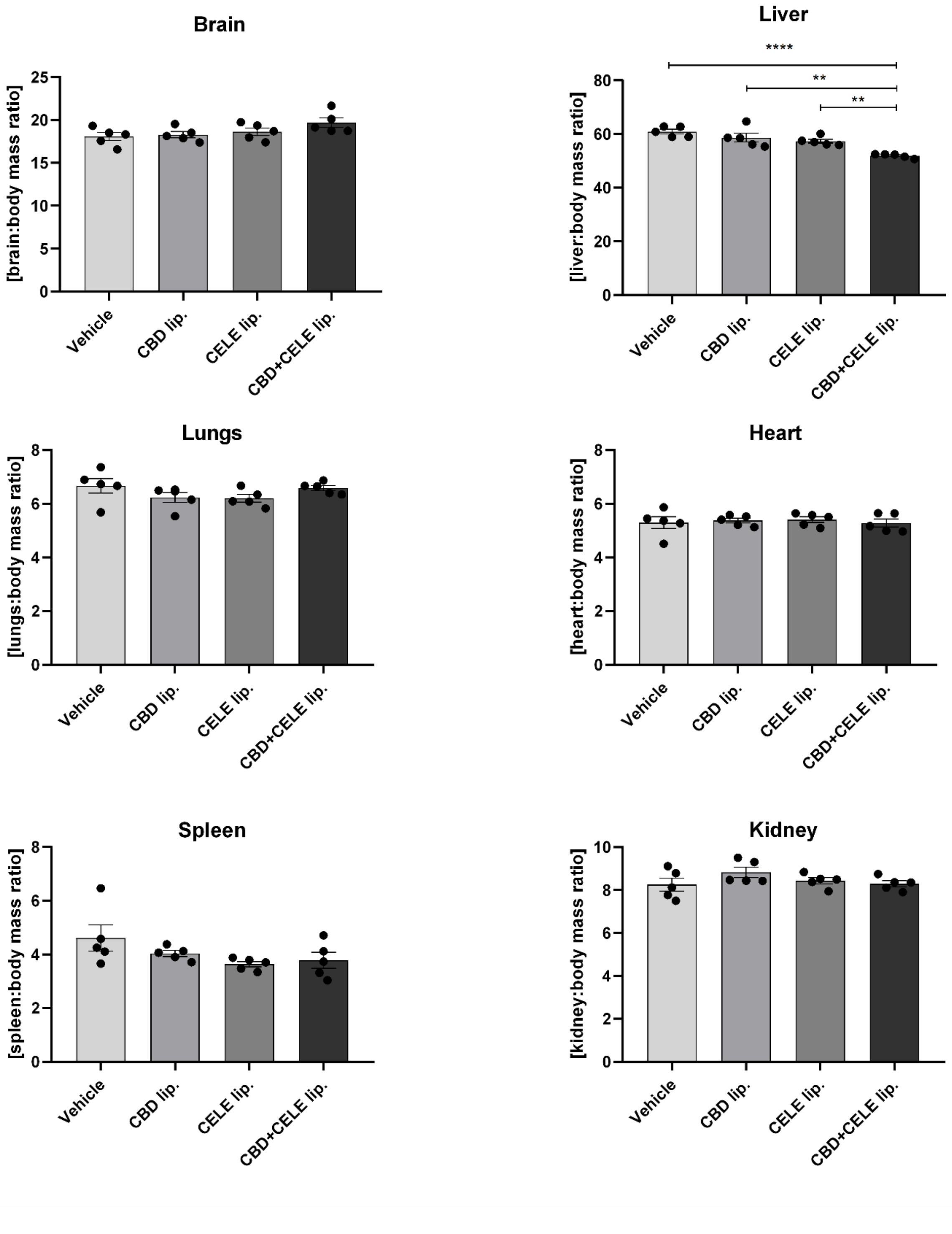

Supplementary Figure S1:

Relative organ weights (organ mass:body mass ratio) of brain, liver, lungs, heart, spleen and kidneys. The measurements were determined at the end of the experiment (day 24) in control and treated mice after termination. Data are shown as mean ± SD. Statistical significant changes between groups were marked by asterisks (* $p<0.05$; ** $p<0.01$; ***$p<0.0001$).
